# Cobalt oxide nanoparticles induce cytotoxicity and excessive ROS mediated mitochondrial dysfunction and p53-independent apoptosis in melanoma cells

**DOI:** 10.1038/s41598-025-85691-y

**Published:** 2025-01-17

**Authors:** Hanan R. H. Mohamed, Basma A. Mohamed, George M. Hakeem, Shahd H. Elnawasani, Maria Nagy, Rawan Essam, Ayman Diab, Gehan Safwat

**Affiliations:** 1 Department of Zoology, Faculty of Science, Cairo University, Giza, Egypt; 2 Faculty of Biotechnology, October University for Modern Sciences and Arts (MSA), 6th of October City, Egypt

**Keywords:** Co_3_O_4_NPs, Melanoma A-375 cells, Oxidative DNA damage, Mitochondrial dysfunction, p53 independent apoptosis, Genetics, Microbiology, Health care, Medical research

## Abstract

Nanotherapy has emerged as a promising strategy for the targeted and efficient treatment of melanoma, the most aggressive and lethal form of skin cancer, with minimized systemic toxicity. However, the therapeutic efficacy of cobalt oxide nanoparticles (Co_3_O_4_NPs) in melanoma treatment remains unexplored. This study aimed to assess the therapeutic potential of Co_3_O_4_NPs in melanoma treatment by evaluating their impact on cell viability, genomic DNA and mitochondrial integrity, reactive oxygen species (ROS) generation and apoptosis induction in melanoma A-375 cells. Our findings demonstrated a concentration-dependent reduction in cell viability upon treatment with five Co_3_O_4_NP concentrations (0.2, 2, 20, 200, and 2000 µg/ml), with an IC50 value of 303.80 µg/ml. Treatment with this IC50 concentration significantly increased ROS generation, induced dramatic DNA damage, and disrupted mitochondrial membrane potential integrity. Flow cytometric analysis revealed apoptosis and necrosis induction following Co_3_O_4_NP exposure at the IC50 concentration value. Results of qRT-PCR analysis demonstrated remarkable dysregulation of apoptotic and mitochondrial genes, including a significant downregulation of apoptotic p53 and mitochondrial ND3 genes and marked upregulation of the anti-apoptotic gene Bcl2. These findings highlight the novel potential of Co_3_O_4_NPs as potent inducers of melanoma A-375 cell death in a concentration-dependent manner through excessive ROS production, genomic instability, mitochondrial dysfunction and dysregulation of apoptotic and mitochondrial gene expression, ultimately promoting apoptosis in A-375 cells. This study thus underscores the potential of Co_3_O_4_NPs as a promising nanotherapeutic candidate for melanoma treatment, warranting further exploration to elucidate their full biological and clinical applicability.

## Introduction

Malignant melanoma is the most dangerous form of skin cancer originating from melanocytes, the cells responsible for pigment production in the skin. Despite its rarity, this type of skin cancer accounts for the majority of skin cancer deaths. Melanoma poses a major challenge to global health due to its high potential for metastasis and mortality^1^. The incidence of melanoma has been steadily increasing worldwide, with a notable rise in developed countries. This increase has been attributed to several factors, including increased exposure to ultraviolet radiation and increased use of tanning beds^2^.

Melanoma, a highly aggressive form of skin cancer, presents several challenges in its detection difficulties and management, particularly due to its potential for early metastasis and resistance to conventional therapies^3^. Despite significant advancements in cancer treatment, including immunotherapy and targeted therapies, melanoma remains a major challenge due to its resistance to conventional therapies and its tendency to metastasize. Current therapies are often ineffective against advanced melanoma or come with severe side effects, presenting a clear **market gap** for new, more effective treatments^4,5^.

The global market for **melanoma therapeutics** is valued at several billion dollars and is expected to grow significantly in the coming years as the demand for innovative treatments increases. However, the market remains underserved in terms of novel therapeutic options that can specifically target aggressive melanoma cells without significant side effects. This opens up significant opportunities for the development of **nanoparticle-based therapies**, which could potentially overcome the limitations of current treatments and provide more targeted, effective, and less toxic alternatives^6,7^.

Nanotherapy has emerged as a promising strategy to address these challenges. By utilizing nanoparticles, researchers aim to enhance drug delivery specifically to melanoma cells, thereby increasing treatment efficacy while reducing systemic toxicity^6^. Nanoparticles can be engineered to improve the bioavailability of therapeutic agents, overcome drug resistance, and provide targeted therapy by accumulating in tumor tissues^7^. This approach not only allows for precise drug delivery but also minimizes damage to healthy tissues, potentially improving overall patient outcomes. As the field of nanomedicine advances, nanotherapy holds significant promise for overcoming the current limitations in melanoma treatment and providing new avenues for more effective and personalized care^6^.

Cobalt oxide nanoparticles (Co_3_O_4_NPs) for example are gaining significant attention in the field of nanomedicine due to their unique physicochemical properties and potential therapeutic applications. These nanoparticles exhibit high surface area-to-volume ratios and distinctive electronic properties, which make them suitable for various applications, including catalysis, energy storage, and biomedical uses. In particular, Co_3_O_4_NPs have shown promise as agents in cancer therapy due to their ability to induce selective cytotoxicity in human cancer cells^8,9^.

The intrinsic properties of Co_3_O_4_NPs, such as their high reactivity and ability to generate reactive oxygen species (ROS), play a crucial role in their cytotoxic effects. These nanoparticles can effectively induce oxidative stress, leading to increased apoptosis in cancer cells while minimizing damage to normal cells^9–11^. This selective cytotoxicity is particularly advantageous in treating aggressive cancers like melanoma, where traditional therapies often fall short due to resistance and severe side effects^12^. However, the therapeutic efficiency of Co_3_O_4_NPs against melanoma cancer has not been fully explored.

Moreover, Co_3_O_4_NPs have been explored for their potential in targeted drug delivery systems and imaging applications. Their ability to be functionalized with various therapeutic agents and imaging probes allows for enhanced specificity and precision in cancer treatment. Despite their promising applications, the safety profile of Co_3_O_4_NPs, including their genotoxicity and long-term effects, requires careful evaluation to ensure their safe use in clinical settings^13,14^.

The research gap that thus addressed in this study lies in the underexplored potential of Co_3_O_4_NPs as a therapeutic agent for aggressive melanoma, such as the A-375 cell line as their use in melanoma treatment, particularly through mechanisms like oxidative stress induction and apoptosis, has not been fully investigated. This gap is critical because Co_3_O_4_NPs, with their unique physicochemical properties e.g. high surface area, catalytic activity), might provide a new approach to treat melanoma more effectively by targeting the cancer cells in a more specific manner. Additionally, the exact mechanisms by which Co_3_O_4_NPs induce cytotoxicity, ROS generation, and apoptosis in melanoma cells remain unclear.

The present study fills this gap by assessing the cytotoxic effects, ROS generation, genomic and mitochondrial DNA integrity, and apoptotic induction in human melanoma A-375 cells. The findings from this study could lead to the development of novel nanotherapeutics that target melanoma with minimal off-target toxicity and reduced side effects, opening new possibilities for melanoma treatment. To achieve this, we used several methods: the Sulforhodamine B (SRB) assay to measure cell viability, the alkaline Comet assay to assess genomic DNA integrity, and 123-Rhodamine dye to evaluate mitochondrial membrane potential. Level of ROS generation and mitochondrial membrane potential integrity were analyzed using 2,7-Dichlorofluorescein diacetate and 123-Rhodamine dyes, respectively. Moreover, quantitative Real-Time PCR (qRT-PCR) was employed to quantify apoptotic gene expression.

## Materials and methods

### Chemicals

The Co_3_O_4_NPs utilized in the current study were acquired in from Sigma Chemical Company (St. Louis, MO, USA) as s black powder with an average particles size of ≤ 50 nm (product No. 637025, CAS No. 1308-06-1). All the remaining chemicals used throughout the experimental procedures were sourced at high molecular grade to maintain consistency and accuracy in our experimental procedures.

### Characterization of Co_3_O_4_NPs

The Co_3_O_4_NPs were characterized using a charge-coupled device diffractometer (XPERT-PRO, PANalytical, Almelo, Netherlands) to obtain their X-ray diffraction (XRD) patterns. Dynamic light scattering (DLS) analysis was performed with a Malvern Zetasizer Nano Series (Malvern Instruments, Westborough, MA, USA), which uses a He-Ne laser (λ = 633 nm, max 5 mW) to assess the particles’ size distribution and Zeta potential. Additionally, the morphology and average particle size of the suspended Co_3_O_4_NPs were examined using transmission electron microscopy (TEM) with a Tecnai G20 Super Twin double-tilt TEM, operating at an accelerating voltage of 200 kV.

### Cell seeding

Human melanoma (A-375) cells were sourced from Nawah Scientific Inc. (Mokatam, Cairo, Egypt) and were cultured in Dulbecco’s Modified Eagle medium (DMEM) containing 10% inactivated fetal bovine serum, 100 units/ml penicillin, and 100 mg/mL streptomycin. The A-375 cells were maintained in an incubator set at 37 °C with 5% CO2 to ensure optimal growth conditions.

### Estimation of Co_3_O_4_NPs cytotoxicity

To assess the Cytotoxicity of Co_3_O_4_NPs and their impact on the viability of melanoma A-375 cells, a Sulforhodamine B (SRB) cytotoxicity assay was conducted in based on a previously established protocol by^15,16^: A 100 µl suspension of cells was cultured in 96-well plates and incubated in complete cultured DMEM medium for 24 h. After incubation, cells were exposed to Co_3_O_4_NPs at concentrations of 0.2, 2, 20, 200 and 2000 µg/ml. Following 72 h of treatment, the cells were fixed, washed with distilled water, and incubated with a 0.4% SRB solution for 10 min in the dark at room temperature. After staining, the plates were washed with 1% acetic acid and left to dry overnight. The protein-bound SRB was then dissolved, and absorbance was measured at 540 nm using a BMG LABTECH^®^-FLUO Star Omega microplate reader (Ortenberg, Germany). The half-maximal inhibitory concentration (IC50) of Co_3_O_4_NPs in melanoma A-375 cells was determined from three replicates using GraphPad Prism software.

### Experimental design

Melanoma A-375 cells were seeded in T25 flasks under optimized conditions, including appropriate culture medium, pH, temperature, and other environmental factors. The cells were then divided into two groups: untreated (control) and treated cells. Control cells received DMSO at a concentration of less than 0.1%. The treated A-375 cells were exposed to Co_3_O_4_NPs at a concentration corresponding to the IC50 value for 72 h. Following treatment, both control and treated A-375 cells were centrifuged, trypsinized, and washed twice with ice-cold PBS. The harvested cells were stored at −80 °C in PBS for future molecular analysis. Triplicates of the Co_3_O_4_NPs treatment were conducted to ensure precision and consistency in the results.

### Evaluation of intracellular ROS generation

The generation of ROS in untreated and Co_3_O_4_NPs-treated melanoma A-375 cells was studied using 2,7-dichlorofluorescin diacetate (DCFH-DA) dye^17^. A suspension of A-375 cells was mixed with DCFH-DA dye (20 mM) and incubated in the dark at room temperature for 30 min. During this time, the dye entered the cells and specifically reacted with ROS forming dichlorofluorescein (DCF), a fluorescent compound. After incubation, the cell-dye mixture was placed on a clean slide and analyzed under an epi-fluorescent microscope at 200× magnification. Images were captured and screened the emitted fluorescent light, an indicator of ROS production in the A-375 cancer cells.

### Estimation of genomic DNA stability

Genomic DNA stability was assessed in both untreated and Co_3_O_4_NPs-treated melanoma A-375 cells using the single-cell gel electrophoresis alkaline comet assay^18,19^. Melanoma A-375 cells were treated with Co_3_O_4_NPs at the IC50 concentration for 72 h. A suspension of A-375 cells (15 µl, ) was added to low-melting agarose (60 µl), gently mixed and spread onto a slide pre-coated with normal-melting agarose (1%). After allowing the gel to solidify, the slides were incubated in a cold lysis buffer containing freshly added DMSO and Triton-X100 for 24 h at 4 °C in the dark. The slides were then placed in freshly prepared alkaline electrophoresis buffer (pH > 12) for 15 min and electrophoresed at 25 V and 300 mA for 30 min. After electrophoresis, the slides were neutralized, fixed, dried, and stained with ethidium bromide for imaging. The resulting comet nuclei, which exhibited varying levels of DNA damage, were analyzed using COMETSCORE™ software. Comet parameters: tail length, %DNA in the tail, and tail moment were reported as mean ± standard deviation (SD).

### Studying the integrity of the mitochondrial membrane potential

The integrity of mitochondrial membrane potential was analyzed in both untreated and Co_3_O_4_NPs-treated melanoma A-375 cells using the fluorescent Rhodamine-123 dye^20^. A suspension of A-375 cancer cells was added to each mixed with Rhodamine-123 fluorescent dye (10 mg/ml), gently mixed and left in the dark for 1 h at 37 °C. The cells were then washed twice with PBS, spread on a clean sterile slide, and visualized using an epi-fluorescence microscope at 200× magnification to screen the fluorescence emitted from Rhodamine-123 stained cells.

### Detection of apoptosis induction

Apoptosis induction was assessed in melanoma A-375 cells following exposure to an IC50 concentration of Co_3_O_4_NPs using Flow Cytometry based on the manufacturer’s protocol for the Annexin V- Fluorescein isothiocyanate (FITC) apoptosis detection kit (Abcam Inc., Cambridge, UK). A dual-channel Flow Cytometer was used to differentiate between apoptotic and necrotic cells. After 72 h of treating A-375 cells with an IC50 concentration of Co_3_O_4_NPs, the cells were collected via trypsinization and washed twice with ice-cold PBS (pH 7.4). The collected cells were incubated in the dark with an Annexin V-FITC/propidium iodide (PI) solution for 30 min at room temperature, and then cells were analyzed using the ACEA Novocyte Flow Cytometer (ACEA Biosciences Inc., San Diego, CA, USA), with FITC and PI fluorescence measured by FL1 and FL2 detectors, respectively (λex/em 488/530 nm for FITC and λex/em 535/617 nm for PI). Each sample underwent acquisition of 12,000 events, and positive FITC and/or PI cells were quantified through quadrant analysis using ACEA NovoExpress software (ACEA Biosciences Inc., San Diego, CA, USA).

### Estimation of apoptotic and mitochondrial gene expression

The mRNA expression level of apoptosis related (p53 and Bcl2) genes and mitochondrial ND3 gene was measured in control and treated melanoma A-375 cells utilizing quantitative Real-Time polymerase chain reaction (qRT-PCR). This was done by extracting the cellular RNA from untreated and Co_3_O_4_NPs-treated cells following the manufacturer’s instructions of Thermo Fisher Scientific’s GeneJET RNA Purification Kit (USA). A 1 µg of the extracted RNA was then reverse transcribed into complementary DNA (cDNA) using the cDNA Reverse Transcription Kit from Applied Biosystems (Foster City, CA, USA). For measuring the mRNA expression levels of p53, Bcl2, and ND3 genes, a separate qRT-PCR was conducted for each sample in the StepOnePlus Real-Time PCR System (Applied Biosystems). To measure the mRNA expression level of the p53, Bcl2, and ND3 genes, a separate qRT-PCR was performed for each sample using the StepOnePlus Real-Time PCR System (Applied Biosystems). Amplification was carried out with SYBER Green PCR Master Mix and the previously designed primer sequences shown in Table 1^21–23^. The expression levels of the target genes were normalized against the housekeeping gene GAPDH, and the comparative Ct (ΔΔCt) method was used to calculate the fold change in gene expression. Results were presented as mean ± SD.

**Table 1:** Sequences of primers used in qRT-PCR.

Gene	Strand	Primer’s sequences
GAPDH	Forward	5′-GAAGGTGAAGGTCGGAGTCA-3′
	Reverse	5′-GAAGATGGTGATGGGATTTC-3′
ND3	Forward	5′-CGCCGCCTGATACTGGCAT-3′
	Reverse	5′-CTAGTATTCCTAGAAGTGAG-3′
BCL-2	Forward	5′-TCCGATCAGGAAGGCTAGAGT-3′
	Reverse	5′-TCGGTCTCCTAAAAGCAGGC-3′
P53	Forward	5′-CAGCCAAGTCTGTGACTTGCACGTAC-3′
	Reverse	5′-CTATGTCGAAAAGTGTTTCTGTCATC-3′

### Statistical analysis

The results from the alkaline Comet assay and qRT-PCR were analyzed with the Statistical Package for the Social Sciences (SPSS) and are reported as mean ± SD. An unpaired Student’s *t*-test was used to compare the treated and untreated cells.

## Results

### Characterization of Co_3_O_4_NPs

**Fig. 1:**
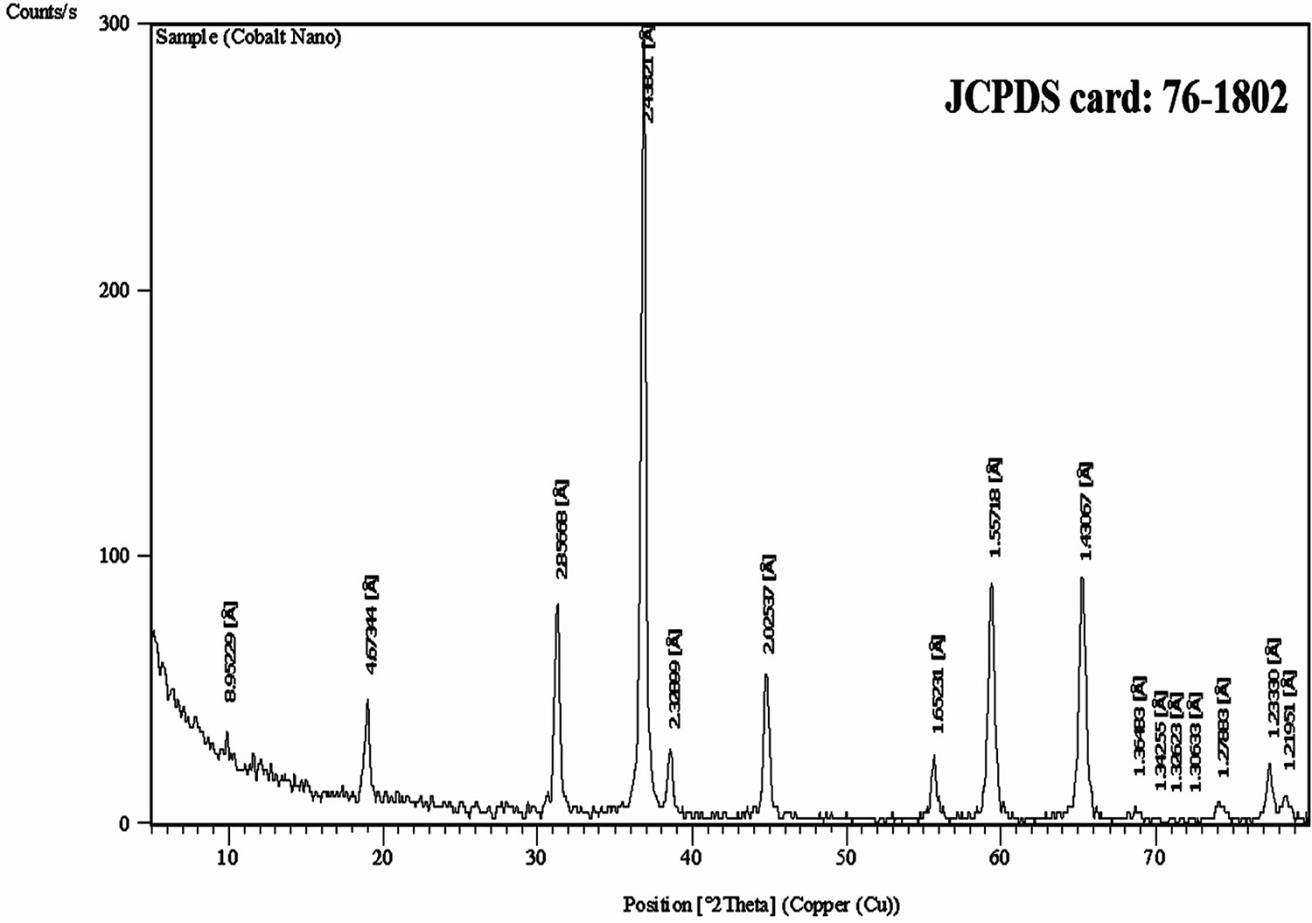
X-ray diffraction (XRD) pattern of Co_3_O_4_NPs.

**Fig. 2:**
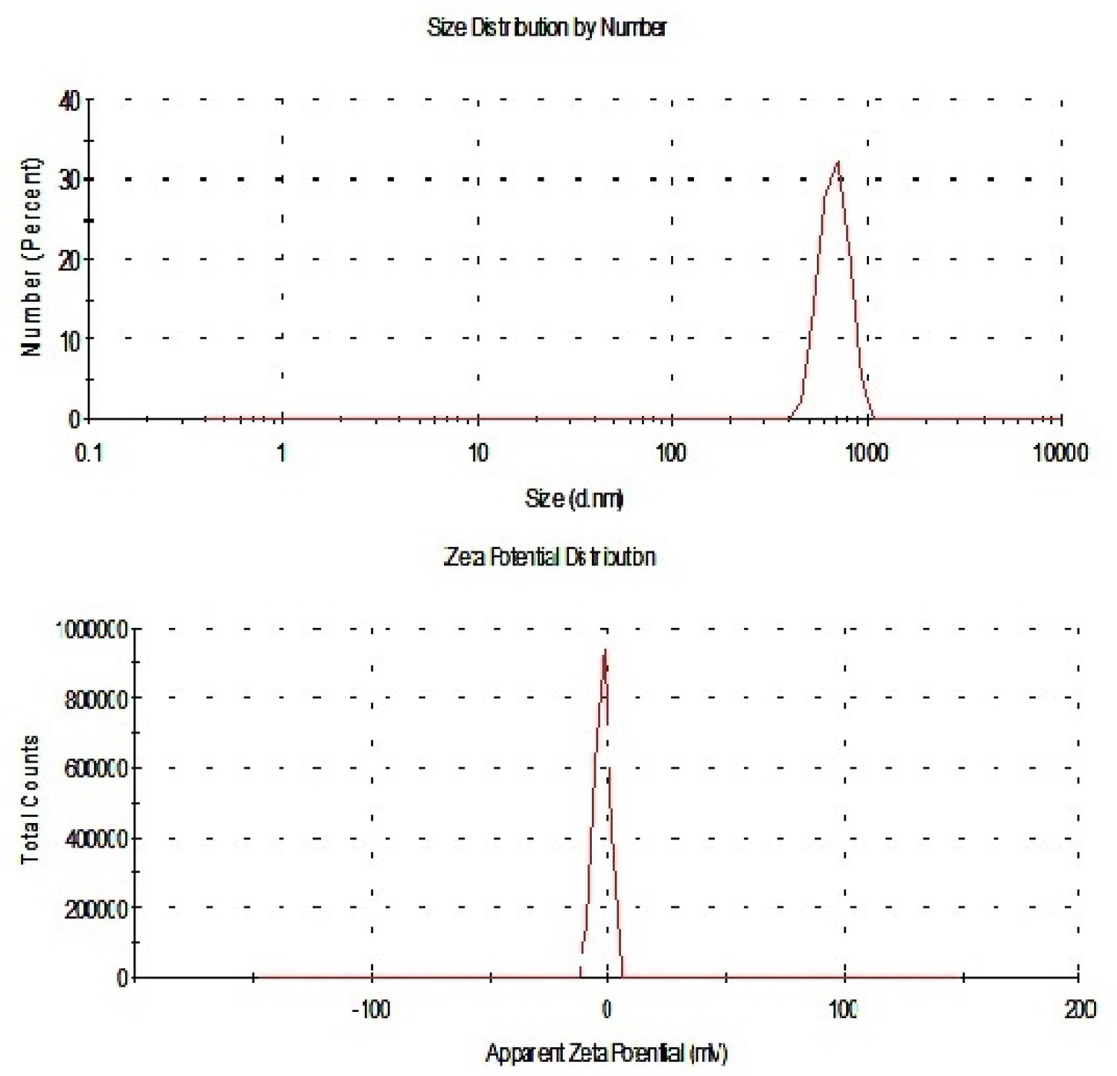
Dynamic laser scattering (DLS) showing the particles distribution and Zeta potential of Co_3_O_4_NPs.

**Fig. 3:**
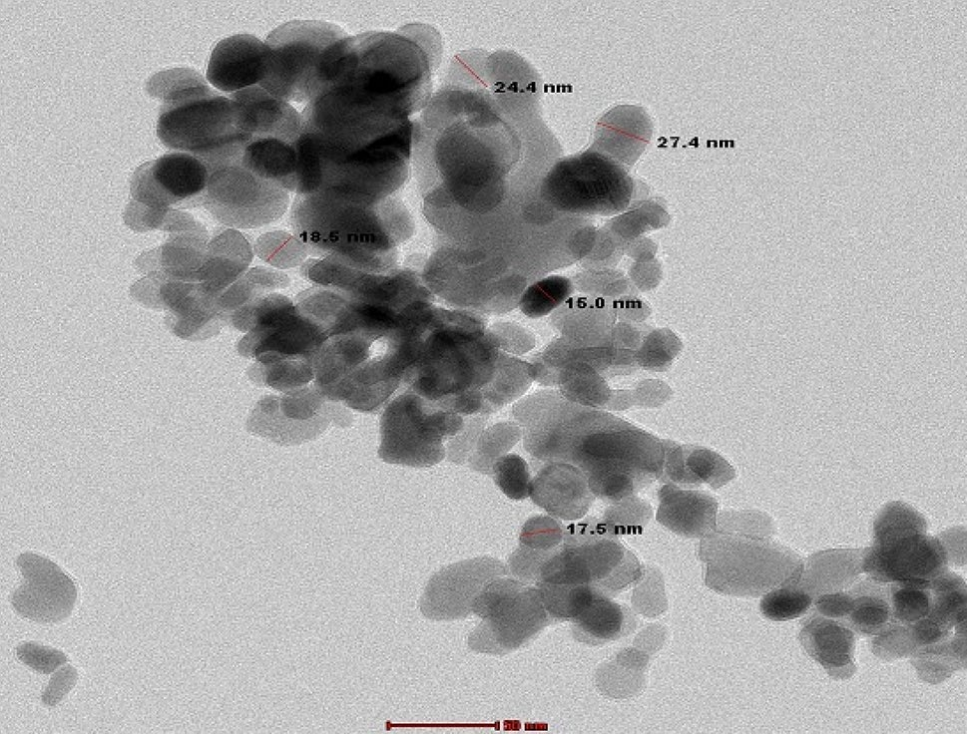
Transmission electron microscope (TEM) imaging of Co_3_O_4_NPs.

Characterization of Co_3_O_4_NPs using XRD analysis confirmed the purity and crystalline structure of Co_3_O_4_NPs through the peaks detected at characteristic diffraction angles of 18.99°, 31.31°, 36.86°, 38.66°, 44.75°, 59.35°, and 65.21° as shown in Fig. 1. Indeed, DLS analysis revealed the well distribution of Co_3_O_4_NPs with an average particle size of 691.7 nm and a Zeta potential value of −2.11 mV, indicating the stability of the Co_3_O_4_NPs in aqueous media (Fig. 2). Imaging of Co_3_O_4_NPs using TEM demonstrated the cubic-spherical shape and well dispersion of Co_3_O_4_NPs with an average particle size of 20.08 as seen in Fig. 3.

### Potent cytotoxicity of Co_3_O_4_NPs

**Fig. 4:**
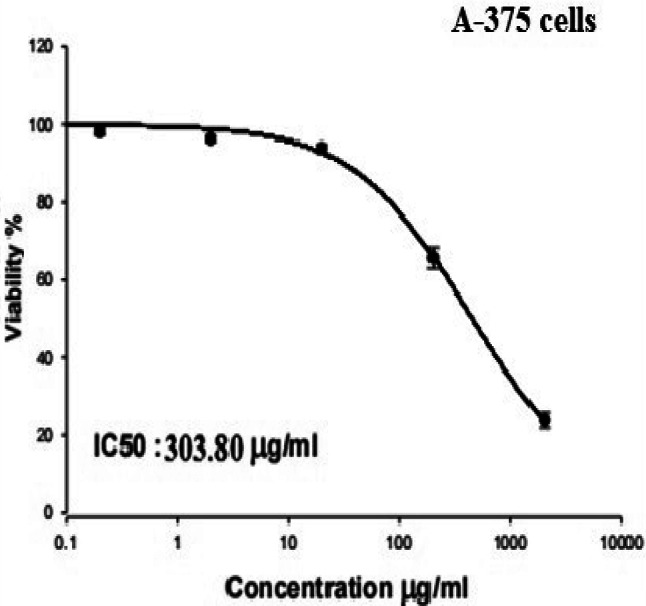
Viability of melanoma A-375 cells following exposure to Co_3_O_4_NPs five concentrations (0.2, 2, 20, 200, 2000 µg/ml) for 72 h.

Interpretation of the cytotoxicity SRB assay results revealed the strong cytotoxicity of Co_3_O_4_NPs on melanoma A-375 cells. This highly toxic effect was manifested through the remarkable concentration-dependent increase in cell death and a corresponding high decrease in cell proliferation noticed after 72 h of melanoma A-375 cells exposure to five different concentrations of Co_3_O_4_NPs: 0.2, 2, 20, 200 and 2000 µg/ml. The IC50 value, at which 50% of the melanoma cells were inhibited, was determined to be 303.80 µg/ml, as seen in Fig. 4.

### Co_3_O_4_NPs over generate ROS

**Fig. 5:**
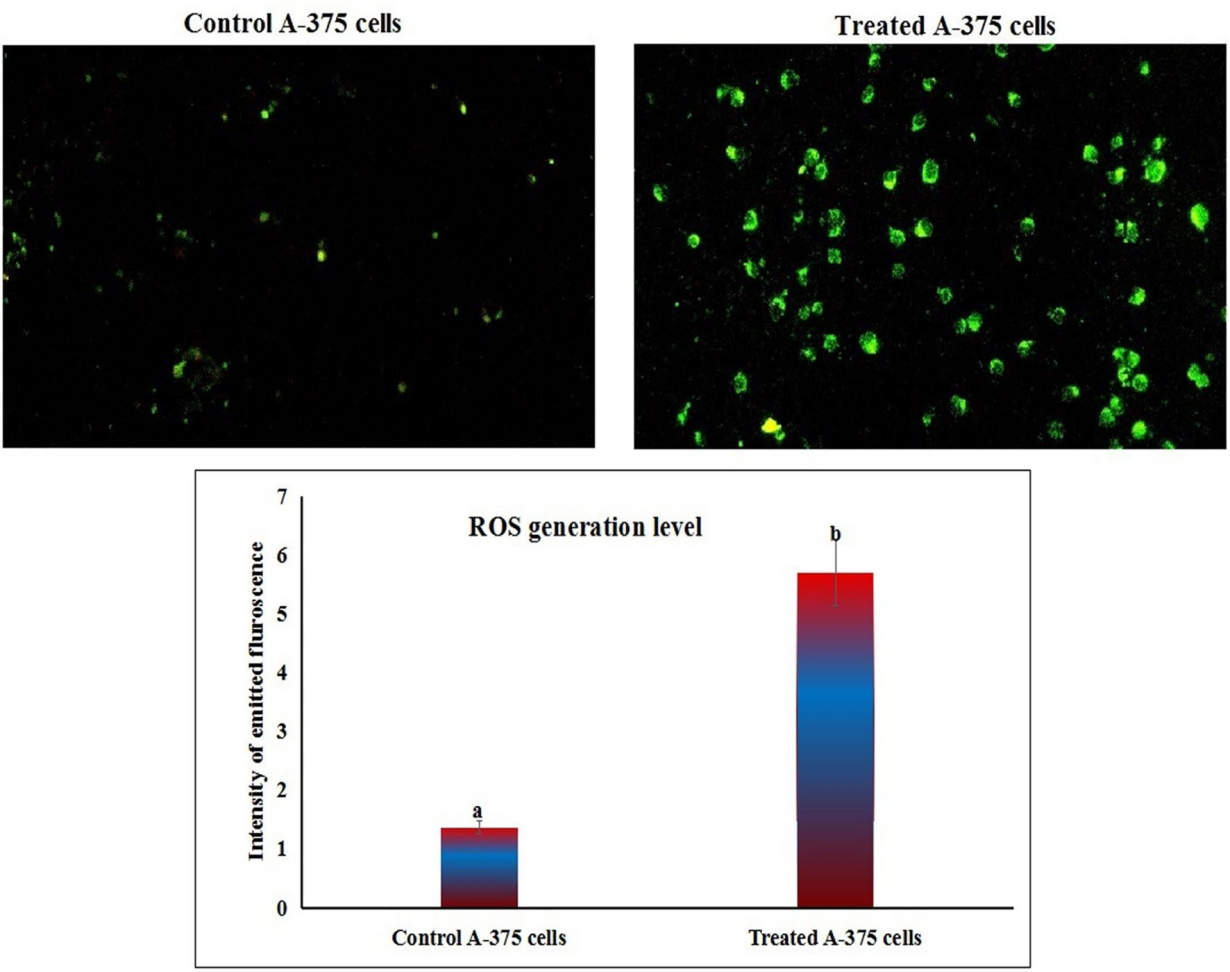
Level of ROS generation within untreated (control) and Co_3_O_4_NPs-treated (303.80 µg/ml) melanoma A-375 cells. Different letters indicate significant difference at *p* < 0.001.

As displayed in Fig. 5, treatment of A-375 melanoma cells with the IC50 concentration of Co_3_O_4_NPs (303.80 µg/ml) led to a significant increase in the production of highly reactive ROS molecules within the A-375 cells. This increase was indicated by the higher intensity of fluorescent light emitted from the CoO NP-treated melanoma A-375 cells compared to the fluorescence from untreated A-375 cells (Fig. 5).

### Co_3_O_4_NPs induce genomic DNA damage

**Fig. 6:**
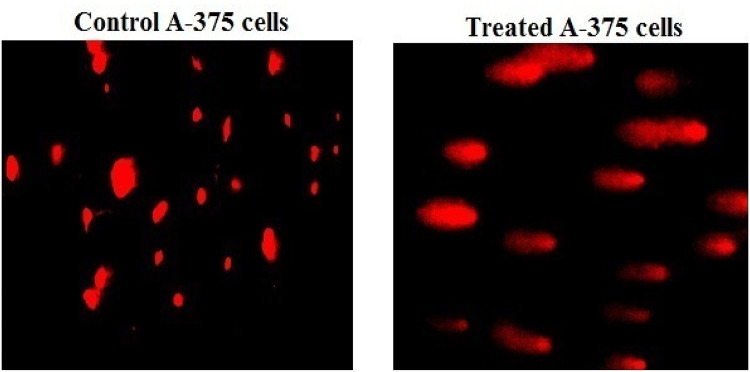
Representative photos for the scored Comet nuclei with intact and damaged DNA following exposure to Co_3_O_4_NPs IC50 concentration (303.80 µg/ml).

Assessment of DNA damage induction using the alkaline Comet assay demonstrated the dramatic induction of genomic DNA damage by Co_3_O_4_NPs in melanoma A-375 cells (Table 2; Fig. 6). This damage was detected by the statistically significant (*p* < 0.01) elevations noticed in the measured DNA damage parameters: tail length, %DNA in tail and tail moment in melanoma A-375 cells treated with 303.80 µg/ml of Co_3_O_4_NPs for 72 h compared to the same measured parameters in untreated A-375 cells (Table 2).

**Table 2:** Integrity of genomic DNA in the melanoma A-375 cells following exposure to IC50 concentration of Co_3_O_4_NPs for 72 h.

Cell	Treatment (µg/ml)	tail length (px)	%DNA in tail	Tail moment
A375 cells	Co_3_O_4_NPs 0.00	6.82 ± 1.51	26.38 ± 1.83	1.83 ± 0.51
	Co_3_O_4_NPs (303.80)	15.01 ± 0.84**	40.83 ± 3.18**	6.36 ± 0.62**

Results are expressed as mean ± SD.

**, ***: Indicates statistical significant difference from the compared untreated control cells at *p* < 0.01 and 0.001, respectively using independent Student *t*-test.

### Co_3_O_4_NPs disrupt the mitochondrial membrane potential

**Fig. 7:**
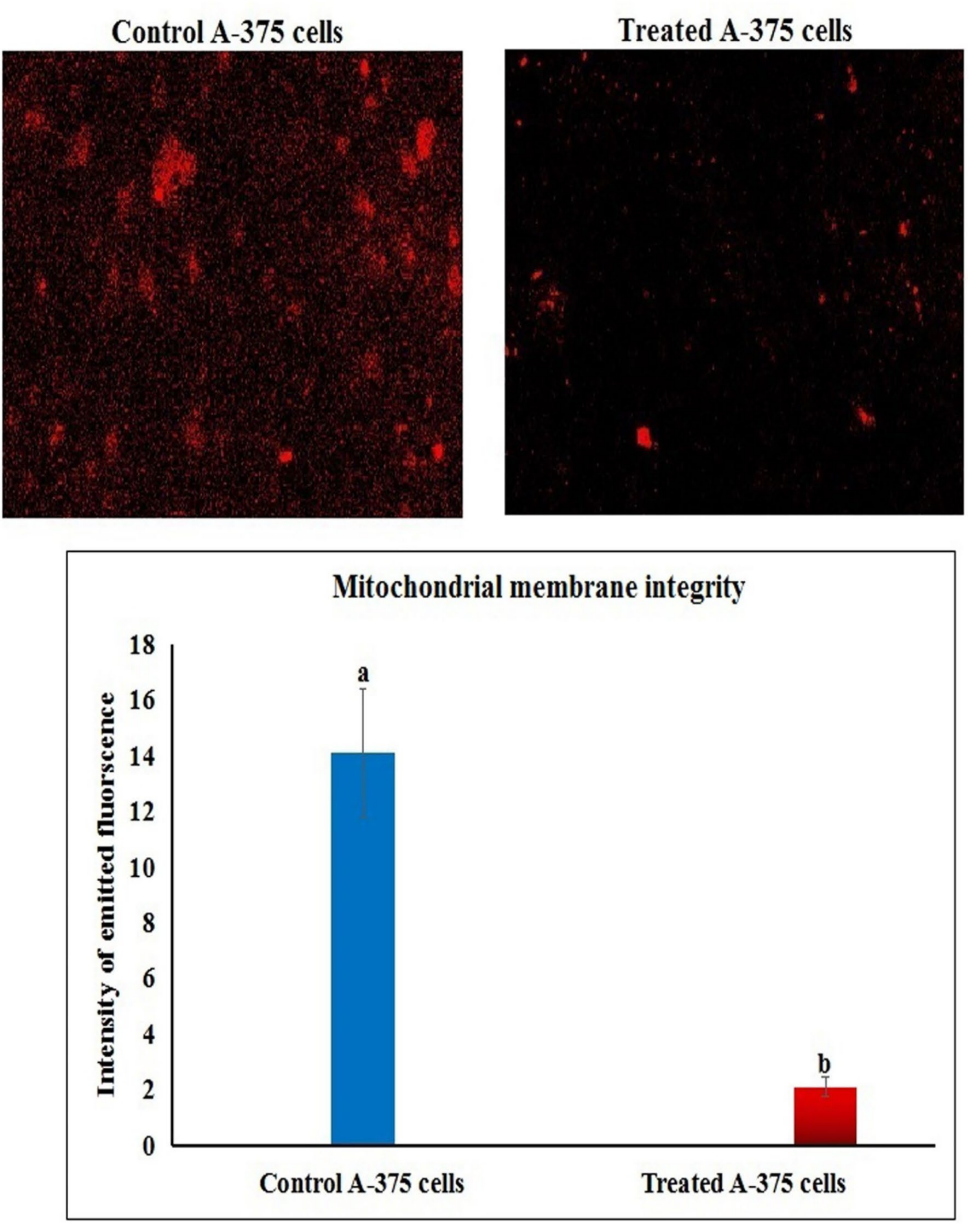
Integrity of mitochondrial membrane potential in the melanoma A-375 cells following exposure to Co_3_O_4_NPs IC50 concentration (303.80 µg/ml) for 72 h. Different letters indicate significant difference at *p* < 0.001.

Fluorescent microscopy of Rhodamine-123-stained melanoma A-375 cells revealed a loss of mitochondrial membrane potential integrity upon exposure to Co_3_O_4_NPs (303.80 µg/ml) for 72 h, as seen in Fig. 7. This was demonstrated through the remarkable decrease noticed in the intensity of fluorescent light emitted from Co_3_O_4_NPs-treated cells compared to untreated A-375 cells (Fig. 7).

### Co_3_O_4_NPs induce apoptosis and necrosis

**Fig. 8:**
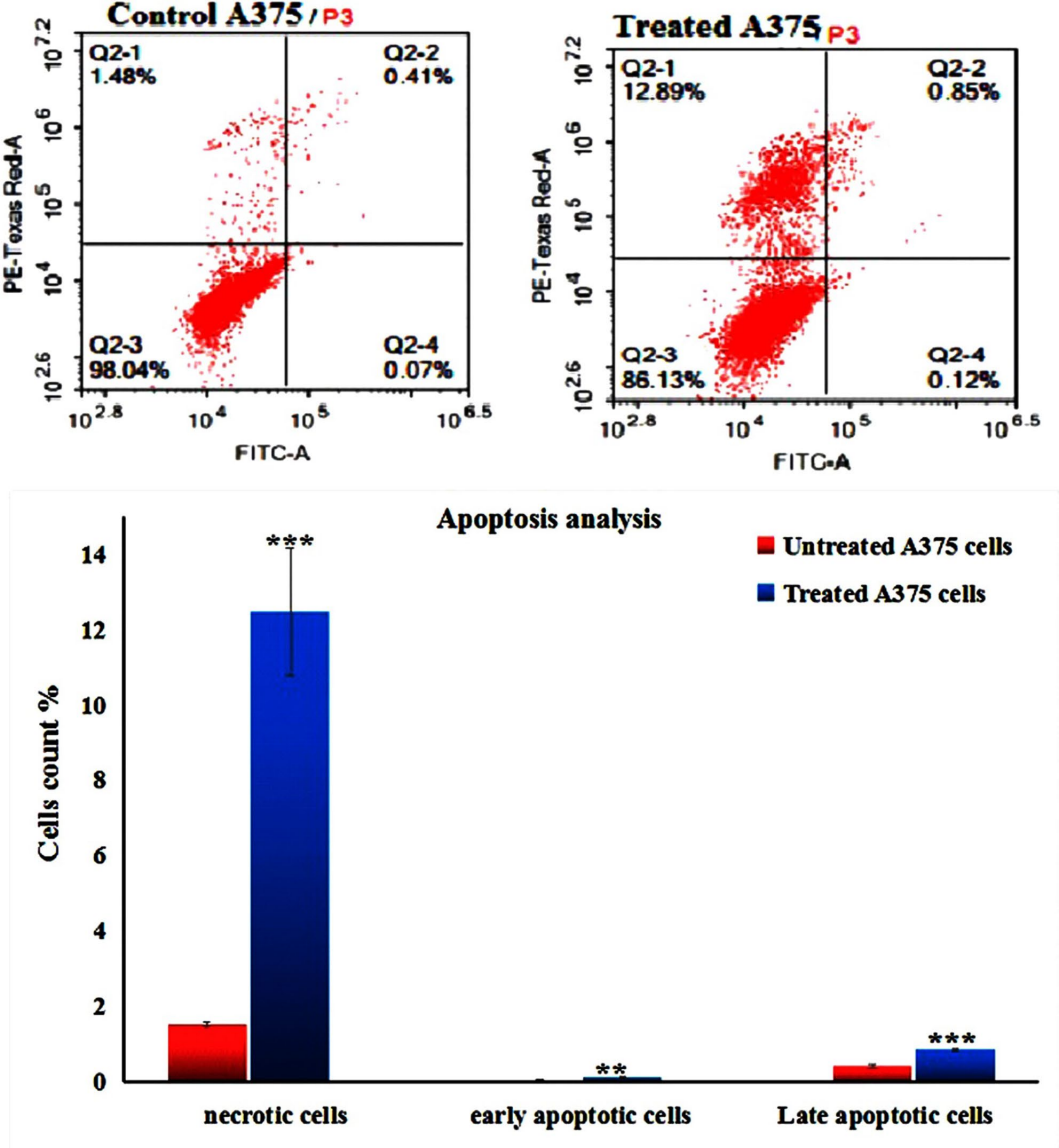
Apoptosis induction in the untreated (control) and treated A-375 cells with an IC50 concentration of Co_3_O_4_NPs (303.80 µg/ml) for 72 h. Q2-1 denotes necrosis phase; Q2-2 denotes late apoptosis phase, Q2-3 denotes normal viable cells and Q2-4 denotes early apoptosis phase.

Flow Cytometric analysis of melanoma A-375 cells demonstrated the induction of apoptosis and necrosis following 72 h of exposure to the IC50 concentration of Co_3_O_4_NPs (303.80 µg/ml) as manifested in Fig. 8 by the statistically significant elevations in the percentage of necrotic (*p* < 0.001), early apoptotic (*p* < 0.01) and late apoptotic (*p* < 0.001) melanoma A-375 cells compared to the untreated A-375 cells.

### Co_3_O_4_NPs dysregulate apoptotic and mitochondrial gene expression

The results of qRT-PCR revealed the remarkable dysregulation of apoptotic and mitochondrial genes expression upon treatment of melanoma A-375 cells with Co_3_O_4_NPs (303.80 µg/ml) for 72 h as seen in Table 3. This dysregulation was demonstrated by the statistically significant decreases in the expression level of apoptotic p53 (*p* < 0.01) and mitochondrial ND3 (*p* < 0.001) genes along with the significant increase (*p* < 0.001) in the expression level of anti-apoptotic Bcl2 gene observed in A-375 cells treated with Co_3_O_4_NPs compared to their expression level in the untreated A-375 cells (Table 3).

**Table 3:** The expression level of p53, ND3 and Bcl2 genes in the melanoma A-375 cells following exposure to IC50 concentration of Co_3_O_4_NPs for 72 h.

Cells	Treatment (µg/ml)	Fold change in the expression level of		
		p53 gene	ND3 gene	Bcl2 gene
A375 cells	Co_3_O_4_NPs 0.00	1.00 ± 0.00	1.00 ± 0.00	1.00 ± 0.00
	Co_3_O_4_NPs (303.80)	0.63 ± 0.07**	0.82 ± 0.02***	4.13 ± 0.15***

Results are expressed as mean ± SD.

**, ***: Indicates statistical significant difference from the compared untreated control cells at *p* < 0.01 and 0.001, respectively using independent Student *t*-test.

## Discussion

Melanoma, a highly aggressive and treatment-resistant form of skin cancer, is known for its rapid proliferation and notable resistance to conventional therapies, making the development of novel treatment strategies essential. One promising candidate for various medical applications, including cancer therapy is Co_3_O_4_NPs due to their unique physicochemical properties. Their small size, high surface area, and catalytic activity make them suitable for diverse biomedical applications, ranging from drug delivery to imaging and photo-thermal therapy. However, the therapeutic potential of Co_3_O_4_NPs specifically against aggressive melanoma has not yet been explored. Therefore, the current study was conducted to estimate the effect of Co_3_O_4_NPs exposure on cell viability, genomic and mitochondrial DNA integrity, ROS generation and apoptosis induction in human melanoma A-375 cells.

The potent cytotoxicity of Co_3_O_4_NPs against the highly aggressive melanoma A-375 cells was demonstrated through the remarkable concentration-dependent increase in cell death and a corresponding decrease in cell proliferation noticed after exposing the A-375 cells to varying concentrations of Co_3_O_4_NPs for 72 h. The IC50 value was determined to be 303.80 µg/ml. These findings align with previous studies that demonstrated strong cytotoxic effects of Co_3_O_4_NPs against other human cancer cells, including hepatocellular carcinoma Hep-G2 cells, CT26 colorectal cancer cells and mouse colon adenocarcinoma cells^9–11^.

To further understand the cytotoxicity induced by Co_3_O_4_NPs on highly aggressive melanoma A-375 cells, we assessed ROS generation, genomic DNA integrity, mitochondrial membrane potential, and apoptosis induction following a 72-hour exposure to the IC50 concentration (303.80 µg/mL) of Co_3_O_4_NPs. Staining with 2, 7-DCFH-DA dye revealed a substantial increase in ROS generation level in Co_3_O_4_NPs-treated A-375 cells compared to untreated controls. The detection of total cellular ROS using 2,7-DCFH-DA dye relies on its oxidation by ROS to form 2,7-dichlorofluorescein (DCF). Once inside the cells, 2,7-DCFH-DA is de-acetylated by cellular esterases, producing 2,7-dichlorodihydrofluorescein (DCFH). ROS then oxidize DCFH to generate DCF, a fluorescent compound that emits green light with an excitation wavelength of 485 nm and an emission wavelength of 530 nm. Consistent with previous studies, these findings suggest that Co_3_O_4_NPs -induced cytotoxicity in A-375 melanoma cells is largely driven by excessive ROS generation, which disrupts cellular homeostasis and damages critical cellular components such as DNA, lipids, and proteins leading to oxidative stress and apoptosis^9–11,24^.

DNA damage is a key consequence of oxidative stress, where excessive ROS can induce various DNA lesions, including single-strand DNA breaks and more severe double-strand DNA breaks^25^. In this study, Co_3_O_4_NPs -induced DNA breaks were confirmed by significant increases in the Comet assay parameters—tail length, %DNA in tail, and tail moment noticed after a 72-hour exposure of melanoma A-375 cells to the IC50 concentration of Co_3_O_4_NPs compared to untreated cells. The DNA breaks detected, including single- and double-strand breaks via the alkaline Comet assay, pose critical threats to cell survival and can initiate apoptosis, as double-strand breaks alone are sufficient to trigger cell death^26^. Consequently, the excessive ROS generation and substantial DNA damage induced by Co_3_O_4_NPs ultimately lead to apoptosis in melanoma A-375 cells.

Apoptosis, or programmed cell death, is a critical process in maintaining cellular homeostasis by regulating the balance between cell growth and death. The regulation of apoptosis involves a complex interplay of pro-apoptotic and anti-apoptotic factors, among which the tumor suppressor protein p53 and the anti-apoptotic Bcl-2 protein play significant roles. In many human cancers, there is a deregulation of this balance. The tumor cells can avoid apoptosis through a loss of balance between anti- and pro-apoptotic proteins, reduced caspase function and impaired death receptor signaling^27,28^. In this study, apoptosis induction by Co_3_O_4_NPs in highly aggressive melanoma A-375 cells was manifested through the statistical significant increases in the number of necrotic and apoptotic A-375 cells noticed 72 h after melanoma A-375 cells exposure to Co_3_O_4_NPs at a concentration of 33.80 µg/ml.

Members of the BCL-2 family have pro- or anti-apoptotic activities. Overexpression of BCL2 gene is generally associated with increased cell survival. However, excessive Bcl-2 gene expression can upset cellular homeostasis, leading to dysregulated apoptosis. Under sustained cellular stress, overexpression of Bcl-2 may paradoxically promote apoptosis through interactions with other members of the Bcl-2 family, and also through cellular mechanisms that bypass Bcl-2’s inhibitory functions. Consequently, elevated BCL2 expression can increase cellular sensitivity to apoptosis, especially in the presence of mitochondrial-targeting stressors^29,30^.

Consistency with this fact Co_3_O_4_NPs induced apoptosis demonstrated in melanoma A-375 cells can be attributed to the noticed concurrent marked upregulation of Bcl2 gene expression alongside with significant down regulation of p53 and mitochondrial ND3.The downregulation of p53 and ND3 gene expression sets up a cellular environment marked by unchecked DNA damage, impaired mitochondrial function, and increased oxidative stress^27,31,32^. The damage to mitochondria was demonstrated in melanoma A-375 cells treated with Co_3_O_4_NPs through the remarkable decreased fluorescence in Rhodamine-123 stained A-375 cells, and could be attributed to the Co_3_O_4_NPs detected excessive ROS generation because over production of ROS cause severe mitochondrial damage leading to additional ROS generation and loss of mitochondrial membrane potential, increasing membrane permeability^33^.

Without p53, cells are also less able to repair DNA or arrest the cell cycle in response to damage, leading to genomic instability. Simultaneously, mitochondrial ND3 gene downregulation weakens mitochondrial function, increasing ROS production and mitochondrial damage. Together, these changes push cells toward apoptosis as the cumulative stress surpasses cellular tolerance, indicating a potential therapeutic approach for targeting cells with dual p53 and ND3 deficiencies^27,31,32^.

## Conclusion

Based on the data presented, Co_3_O_4_NPs exhibit strong, concentration-dependent cytotoxicity against the highly aggressive melanoma A-375 cells through a cascade of cellular damage that severely affected cell viability and function. Exposure to the IC50 concentration of Co_3_O_4_NPs resulted in excessive ROS generation, which disrupted mitochondrial membrane potential and integrity. This mitochondrial dysfunction, coupled with elevated ROS generation, led to substantial DNA damage, upregulated Bcl2 gene expression and downregulated p53 and ND3 gene expression impairing the cell’s ability to repair itself. Together, these effects increased the cells’ susceptibility to apoptosis and ultimately promoted apoptotic cell death. These findings thus underscore the multifaceted and deleterious impact of Co_3_O_4_NPs on aggressive melanoma A-375 cells, highlighting the need for further studies to assess the possibility of using Co_3_O_4_NPs in melanoma therapy.

## Data Availability

The datasets used and/or analyzed during the current study are available from the corresponding author on reasonable request.
